# Deubiquitylating Nanog: novel role of USP21 in embryonic stem cell maintenance

**DOI:** 10.1038/sigtrans.2017.14

**Published:** 2017-04-21

**Authors:** Duanqing Pei

**Affiliations:** 1 Key Laboratory of Regenerative Biology, Guangdong Provincial Key Laboratory of Stem Cell and Regenerative Medicine, South China Institute for Stem Cell Biology and Regenerative Medicine, Guangzhou Institutes of Biomedicine and Health, Chinese Academy of Sciences 510530, Guangzhou

## Abstract

Recently, three groups independently identified ubiquitin-specific peptidase 21 (USP21) as an efficient deubiquitylase that reverses Nanog polyubiquitylation and stabilizes Nanog protein. In this preview, I have summarized the work of these three groups.

## Main text

How cells integrate a myriad of signals both intrinsic and extrinsic to decide their fates remains a mystery. Pluripotent stem cells or PSCs represent a good model system to unlock part of the secrets. Specifically, embryonic stem cells from human and mouse used to be notoriously difficult to culture until the identification of LIF or leukemia inhibitory factor as a critical ingredient in the media. Interestingly, by screening genes that can alleviate the dependence on LIF, two groups identified Nanog, a transcription factor that can maintain mouse embryonic stem cells (mESCs) at pluripootent state without LIF.^1,2^ Nanog turns out to be part of a network of transcription factors that maintain ESCs at the pluripotent state.

Nanog plays an essential role in the transcriptional network of pluripotency and early embryonic development,^1,2^ controlling the epiblast versus primitive endoderm decision in the blastocyst.^3^ Additional levels of ESC-specific regulation have been characterized, including important roles for TFs and epigenetic regulators. Previous studies have demonstrated that Oct4 and Sox2 are the main transcriptional regulators of Nanog expression in ESCs.^4^ In addition, epigenetic factors, including Wdr5, Mof and Ezh2, can modulate Nanog transcription in ESCs.^5–7^ It is notable that Nanog is a short-lived protein and quickly degraded by the ubiquitin-dependent proteasome system. In this regard, recent study showed that Nanog can be polyubiquitylated by the E3 ubiquitin ligase, F-box and WD40 domain-containing protein 8 (FBXW8), and then degraded, resulting in ESC differentiation.^8^ Interestingly, FBXW8 binding to Nanog requires the phosphorylation of Nanog at N-terminal Serines 52, 71 and 78 by the kinase ERK1. However, little is known about the mechanism and function of the deubiquitinating enzymes that control the protein levels of Nanog in ESC maintenance and differentiation.

Recently, three groups independently identified ubiquitin-specific peptidase 21 (USP21) as an efficient deubiquitylase that reverses Nanog polyubiquitylation and stabilizes Nanog protein.^9–11^ USP21 has been previously demonstrated to deubiquitylate both the nuclear and cytoplasmic proteins, such as GATA3, RIPK1, RIG-1 and Tip5.^12–15^ USP21 functions as a negative regulator in antiviral responses through binding and deubiquitylating RIG-1 in the cytosol.^14^ USP21 also affects the transcription of NF-κB p65 through deubiquitylating and stabilizing interleukin-33 in the nucleus.^13^ USP21 also can stabilize FOXP3 protein and control Treg signature genes.^16^ Moreover, USP21 regulates centrosome- and microtubule-associated functions.^17^ Recent study demonstrated that USP21 recruits and stabilizes Gli1 at the centrosome which is the key transcription factor responsible for Hedgehog (Hh) signaling pathway.^18^ Furthermore, USP21 binds to the promoter region of interleukin-8 and mediates transcriptional initiation and contributes to maintenance of cancer stem cells in Renal cell carcinoma.^19^ Interestingly, USP21 does not only remove ubiquitin from ubiquitylated proteins but also degrades conjugates of the ubiquitin-like protein ISG15 and according to some reports NEDD8.^20,21^

A study from Ping Wang and his colleagues, published in *Nature Communications,* screened 46 mammalian DUBs with a reporter gene system, in which they fused firefly luciferase to the C-terminus of Nanog (Nanog-Luc) to monitor Nanog stability. They found that coexpression with USP21, but not the other DUBs, significantly increased the luciferase activity of Nanog-Luc. They further demonstrated that USP21 prevents the degradation of Nanog through deubiquitylation and thus promote maintenance of embryonic stem cells (ESCs).^9^ Meanwhile, two other labs also report that USP21 regulate the K48-linked polyubiquitination of Nanog.^10,11^ Lingqiang Zhang and his colleagues overexpressed the ectopic USP and OTU subfamilies of DUBs and Nanog in the HEK293T cells and analyzed the expression of Nanog by western blot. Through the screen they also found that USP21 significantly upregulated Nanog levels while other DUBs had little to no effect on the Nanog expression levels.^10^ In both studies, the authors identified USP21 as a specific deubiquitylase for Nanog, but not for Oct4 or Sox2. USP21 interacts with Nanog protein *in vivo* and *in vitro*. The C-terminal USP domain of USP21 and the C-domain of Nanog are responsible for this interaction. During ESC differentiation, USP21 together with Nanog, are downregulated. They also demonstrate that loss of USP21 results in Nanog degradation, mESCs differentiation^9–11^ and reduces somatic cell reprogramming efficiency,^9^ indicating a novel role of USP21 in control of the balance between stem cell maintenance and differentiation. In addition, a most recent study from Kwang–Hyun Baek’s group also identified USP21 as a DUB for Nanog through the yeast two-hybrid sceen of USP subfamily of DUBs, and confirmed the interaction through co-immunoprecipitation and GST pull-down assays. However, in this study, the physiological significance of the interaction has not been investigated.^11^

**Figure 1:**
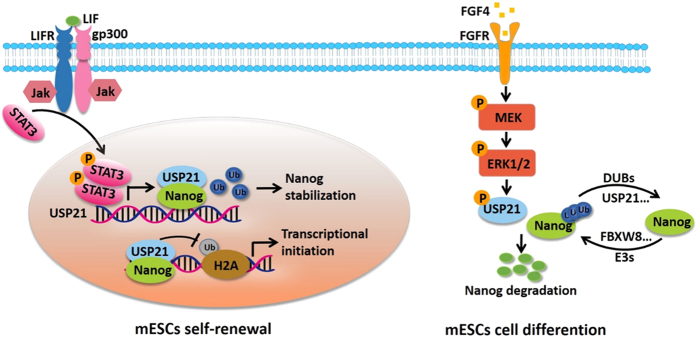
Model showing that USP21 plays an important role in the maintenance of mouse embryonic stem cell (mESC) self-renewal through regulating ubiquitination of Nanog and ubH2A. USP21 is regulated at both transcriptional and post-translational levels in mESC to regulate Nanog function. At the transcriptional level, the expression of USP21 in mESCs was activated by the LIF/STAT3 pathway, which was critical for the maintenance of mESC and the self-renewal of mESCs. Upon mESC differentiation, the expression of USP21 was significantly downregulated. At the post-translational level, USP21 was phosphorylated by ERKs induced by differentiation cues. Phosphorylated USP21 block the interaction with Nanog and accelerate the degradation of Nanog.

Moreover, Ping Wang’s group eludicated the molecular mechasnism of USP21 downregulation during differentiation and found that USP21 is regulated at both transcriptional and post-translational levels in mESC to regulate Nanog function.^9^ At the transcriptional level, the expression of USP21 in mESCs was activated by the LIF/STAT3 pathway, which was critical for the maintenance of mESC and the self-renewal of mESCs. Upon mESC differentiation, the expression of USP21 was significantly downregulated. At the post-translational level, USP21 was phosphorylated by ERKs induced by differentiation cues. This phosphorylation reduced the binding of USP21 to Nanog and led to Nanog degradation. These data suggest that regulation of Nanog by USP21 is a precise and important event to determine the mESC fate (Figure 1).

In addition, USP21 can regulate transcriptional initiation through catalyzing the hydrolysis of the ubiquitylation of histone H2A (H2AK119ub), which represses the di- and trimethylation of H3K4 and epigenetically activate transcriptional activity of target genes.^22^ H2A ubiquitination and chromatin compaction combine to mediate the polycomb repressive complex 1-dependent repression of genes that are crucial for the maintenance of ESC identity.^23^ However, limited information is available concerning the role of USP21 in the regulation of histone function and gene expression. Ping Wang’s group demonstrated that USP21 not only deubiquitinated Nanog by direct interaction but also regulated H2A ubiquitination through being recruited to histones by Nanog (Figure 1).

**Figure 2:**
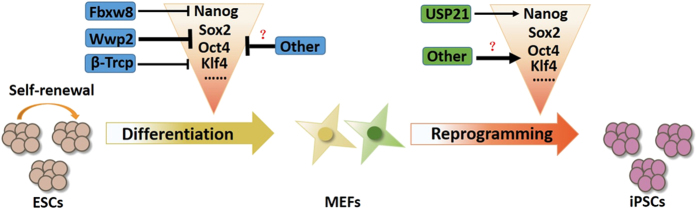
Ubiquitylation regulates embryonic stem cells (ESCs) pluripotency, differentiation and induced pluripotent stem cell (iPSCs) generation. The E3 ligases Fbxw8, Wwp2 and (3-Trcp regulate core transcription factor, such as Nanog, Sox2, Oct4 and Klf4, abundance and functions in ESCs. The deubiquitinating enzymes, USP21 regulate Nanog ubiquitination, maintain the self-renewal of ESCs and promote the efficiency of iPSCs.

On the basis of these and previous studies, it seems that each SCTF is regulated by both ubiquitination and deubiquitination at the post-translational level in a dynamically balanced manner (Figure 2). The net balance of the ubiquitination and deubiquitination of SCTFs could have a significant impact on the cell fate determination of stem cells. Increased ubiquitination of SCTFs leads to degradation and induces cell differentiation, whereas dominant deubiquitination of SCTFs stabilizes these TFs, thus promoting the maintenance of stem cells. These studies also suggest that the protein stability of each SCTF might be controlled by at least a pair of specific DUB and ubiquitin E3 ligase. More and more studies show that SCTFs, such as Nanog, Sox2, c-Myc and Oct4, play an important role in the maintenance of self-renewal of cancer stem cells.^24–27^ Therefore, dissecting this paradigm of reciprocal post-translational control, especially ubiquitination and deubiquitination, in stem cell regulatory networks not only advances stem cell biology but also promotes our understanding of cancer stem cells.
